# Quantification of Degradation Products Formed during Heat Sterilization of Glucose Solutions by LC-MS/MS: Impact of Autoclaving Temperature and Duration on Degradation

**DOI:** 10.3390/ph14111121

**Published:** 2021-11-01

**Authors:** Sarah Leitzen, Matthias Vogel, Michael Steffens, Thomas Zapf, Christa Elisabeth Müller, Martin Brandl

**Affiliations:** 1 Department of Physics, Chemistry and Pharmacy, University of Southern Denmark, 5230 Odense, Denmark; sarah.leitzen@bfarm.de; 2 Federal Institute for Drugs and Medical Devices, 53175 Bonn, Germany; matthias.vogel@bfarm.de (M.V.); michael.steffens@bfarm.de (M.S.); thomas.zapf@bfarm.de (T.Z.); 3 PharmaCenter Bonn, Pharmaceutical Institute, Pharmaceutical & Medicinal Chemistry, University of Bonn, 53121 Bonn, Germany; christa.mueller@uni-bonn.de

**Keywords:** F0 concept, steam sterilization, sterilization safety, glucose degradation products, α-dicarbonyl compounds, derivatization, tandem mass spectrometry, *Geobacillus stearothermophilus*

## Abstract

Heat sterilization of glucose solutions can lead to the formation of various glucose degradation products (GDPs) due to oxidation, hydrolysis, and dehydration. GDPs can have toxic effects after parenteral administration due to their high reactivity. In this study, the application of the F0 concept to modify specific time/temperature models during heat sterilization and their influence on the formation of GDPs in parenteral glucose solutions was investigated using high-performance liquid chromatography-tandem mass spectrometry (LC-MS/MS). Glucose solutions (10%, *w*/*v*) were autoclaved at 111 °C, 116 °C, and 121 °C for different durations. The GDPs glyoxal, methylglyoxal, glucosone, 3-deoxyglucosone/3-deoxygalactosone, 3,4-dideoxyglucosone-3-ene, and 5-hydroxymethylfurfural were quantified after derivatization with o-phenylenediamine by an optimized LC-MS/MS method. For all GDPs, the limit of detection was <0.078 μg/mL, and the limit of quantification was <0.236 μg/mL. The autoclaving time of 121 °C and 15 min resulted in the lowest levels of 3-DG/3-DGal and 5-HMF, but in the highest levels of GO and 2-KDG. The proposed LC-MS/MS method is rapid and sensitive. So far, only 5-HMF concentrations are limited by the regulatory authorities. Our results suggest reconsidering the impurity limits of various GDPs, especially the more toxic ones such as GO and MGO, by the Pharmacopoeias.

## 1. Introduction

**Figure 1:**
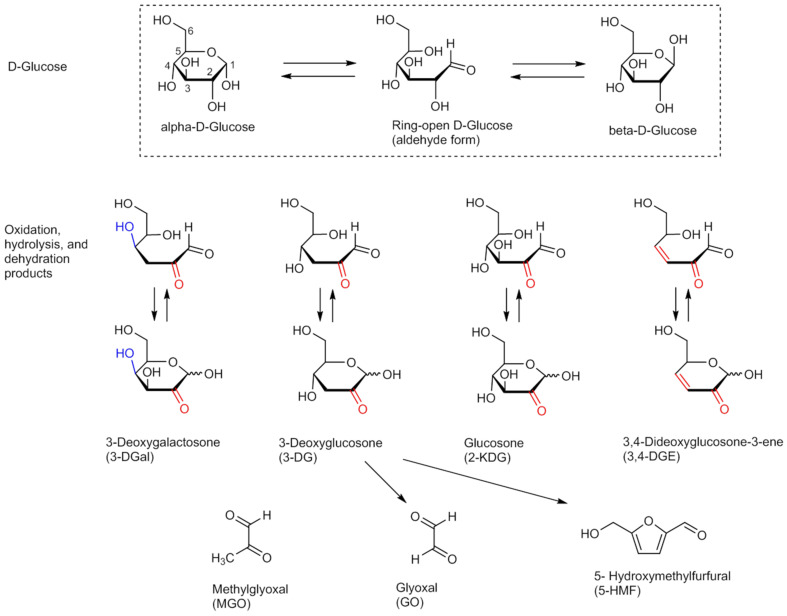
Reaction products of d-glucose due to oxidation, hydrolysis, and dehydration reactions observed during autoclaving of aqueous glucose solutions.

Sterile glucose infusion solutions for parenteral administration are commonly used as reconstitution solvents or diluents for injectable drugs and for peritoneal dialysis [1]. The sterility of the parenteral glucose solutions is a crucial prerequisite for their safety. Sterility can be achieved via several methods and conditions. Whenever possible, a process is chosen in which the product is sterilized in its final container (final sterilization). In order to guarantee the sterility of steam-sterilized glucose solutions, the European Pharmacopoeia stipulates that the products to be steam-sterilized must be heated to at least 121 °C for 15 min (reference sterilization procedure). Other combinations of time and temperature may be used if they achieve a sterility assurance level (SAL) of 10^−6^ or less. This process is to be controlled with the guide germ *Geobacillus stearothermophilus* [2]. The F0 concept is considered to be an equivalent sterilization process; this refers to processes that achieve a comparably lethal effect with different temperature and time combinations as the reference sterilization process described in the European Pharmacopoeia. Sterilization procedures carried out according to the F0 concept have the advantage that sterilization temperatures below 121 °C may be more suitable for temperature-sensitive products and containers offering comparable effects when combined with extended sterilization times [3]. However, reducing the temperature results in a longer autoclaving time. F0 indicates the specified time in minutes to which the solution to be autoclaved is exposed in its final container [4]. During the heat sterilization process of glucose solutions, glucose degradation products (GDPs) can be formed [5,6,7]. So far, some monocarbonyl as well as dicarbonyl degradation products have been identified [8,9,10]. Most GDPs are formed by oxidative and dehydrative processes (Figure 1).

Figure 1 shows the potential GDPs of d-glucose that can be formed by oxidation, hydrolysis, and dehydration.

These are promoted by the hydrolytic activity of the aqueous solvent and by heat. It has been shown that high levels of GDPs may result in the formation of Advanced Glycation End products (AGEs) that have an impact on cellular homeostasis and health in general [1]. Parenteral administration of glucose solutions is expected to lead to an accumulation of these AGEs on the walls of blood vessels [11].

To date, only a few studies have been published that have focused on reducing the formation of GDPs in heat sterilized glucose solutions for parenteral use by the application of appropriate sterilization procedures [12,13,14] and determining the quality and quantity of GDPs in these solutions [1,5,15,16,17].

Due to the frequent use of glucose infusion solutions in practice [1,5] and the discussed possible toxicity of some GDPs such as glyoxal (GO) [18] and methylglyoxal (MGO) [19], this work deals with a current and highly sensitive topic. It is particularly important to closely examine glucose solutions for parenteral administration [11] and to reduce the amount of GDPs generated during the heat sterilization process to a minimum using appropriate methods such as the F0 concept.

In order to investigate parameters that influence heat sterilized glucose solutions potentially leading to different concentrations of formed GDPs such as the α-dicarbonyl (α-DC) compounds GO, MGO, glucosone (2-KDG), 3-deoxyglucosone (3-DG), 3-deoxygalactosone (3-DGal), 3,4-dideoxyglucosone-3-ene (3,4-DGE), and the monocarbonylic compound 5-hydroxymethylfurfural (5-HMF), 10% (*w/v*) glucose solutions in water were prepared. These were heat sterilized using the F0 concept at adapted temperature/time ratios in final containers, and the GDP concentrations were subsequently quantified by LC-MS/MS. In addition, a validation of the method developed for the quantitative measurements was carried out. The success of the autoclaving process was controlled in terms of the inactivation of the bacterial spores of *Geobacillus stearothermophilus*, which is used as a typical key germ.

## 2. Results

Since the 10% (*w*/*v*) glucose solutions are of particular interest with regard to their use as carrier solutions for electrolytes and drugs, we wanted to examine these solutions in more detail [20]. The objective of this study was to show the influence of the heating time when the required autoclaving times (scheme A) are not exactly observed and the glucose solutions are heated for too long (scheme B) (Table 1).

The basis for calculating the sterilization cycles according to schemes A and B can be found in the methods Section 4.2.2.

It is of enormous importance to validate the autoclaving procedure well, especially when working with temperature-sensitive substances such as glucose. For this purpose, an LC-MS/MS method according to Mittelmaier et al. [8], but further modified and optimized, was used to identify and quantify major GDPs in form of α-DCs, in particular GO, MGO, 2-KDG, 3-DG, 3-DGal, 3,4-DGE, and 5-HMF from freshly autoclaved glucose solutions.

### 2.1. Autoclaving under Germicidal Control

This experiment demonstrated that all temperature/time combinations presented by means of the F0 concept in Table 1 were suitable in practice to kill the lead germ *Geobacillus stearothermophilus* used in steam sterilization in the prepared 10% (*w*/*v*) glucose solutions described in Section 4.2.1. If the autoclaving time or temperature had not been sufficient to kill the germ, the germ would have survived the sterilization and secreted acid metabolites in the culture medium after incubation, causing a color change from purple to yellow. This color change did not happen, which can be seen in Figure S1 in the Supplementary Materials. As a control, a non-autoclaved EZ-Test^®^ was co-incubated at 60 °C for 24 h. The test was incubated in the nutrient medium. The “Test/Control” figure clearly shows the color change of the indicator bromocresol purple from purple, at a pH of 6.8 to yellow at more acidic pH values around 5.2 [21]. The results of the autoclaving procedure are additionally described in Table 2.

### 2.2. Measuring of the pH Values of the Autoclaved and Non-Autoclaved Glucose Solutions

The solutions listed in Table 3 were all cooled down to room temperature after heat sterilization, which took place at 111 °C, 116 °C, and 121 °C, and then the pH of these solutions was determined at room temperature.

In Table 3 it can clearly be seen that with increasing heat sterilization temperature from 111 °C to 121 °C, the pH value decreases. In the non-autoclaved glucose solution, the pH value is clearly higher than in the autoclaved one. This can be explained by the fact that the amount of acidic GDPs formed during heat sterilization increases with increasing exposure time and with increased uptake of CO_2_ from the ambient air. The effect of the temperature increase during heat sterilization does not seem to contribute significantly to a reduction of the pH value. Compared to the 10% (*w*/*v*) glucose solution, the pH values of autoclaved water are higher, even those of the non-autoclaved solutions.

### 2.3. Content of GDPs in Autoclaved 10% (*w*/*v*) Glucose Solutions in PP Bottles

Next, we determined the GDP contents in 10% (*w*/*v*) glucose solutions in PP bottles that had been prepared and autoclaved within this study (*n =* 27 measurements each within scheme A/B; for each temperature, 3 bottles per autoclave run were autoclaved in 3 autoclave runs. Each of these 9 bottles was measured 3 times in total). The results are shown in Table 4.

The results of autoclaving the glucose solutions according to scheme A show that the concentrations of GDPs formed decrease with increasing temperature, except for GO and 2-KDG and 3,4-DGE. In comparison, the highest concentrations of GDPs formed are present for 3-DG/3-DGal, 3,4-DGE, and 5-HMF.

**Figure 2:**
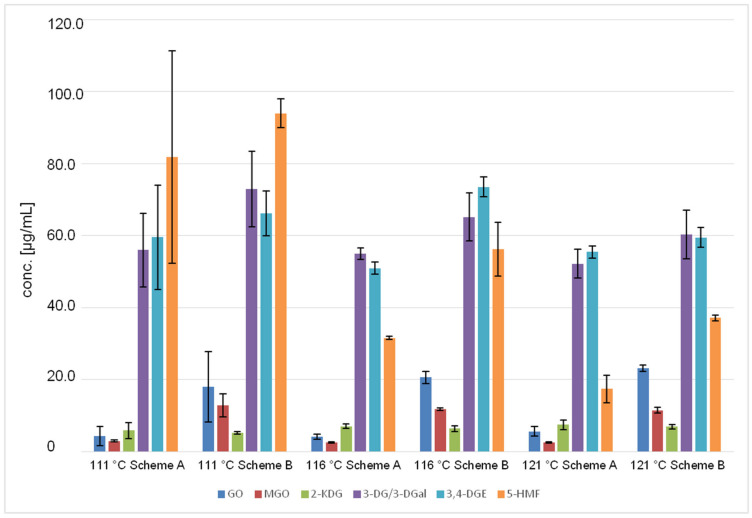
Comparison of the concentrations of GDPs according to the autoclaving scheme (**A**/**B**) and temperature (111 °C, 116 °C, 121 °C) (*n* = 27).

**Figure 3:**
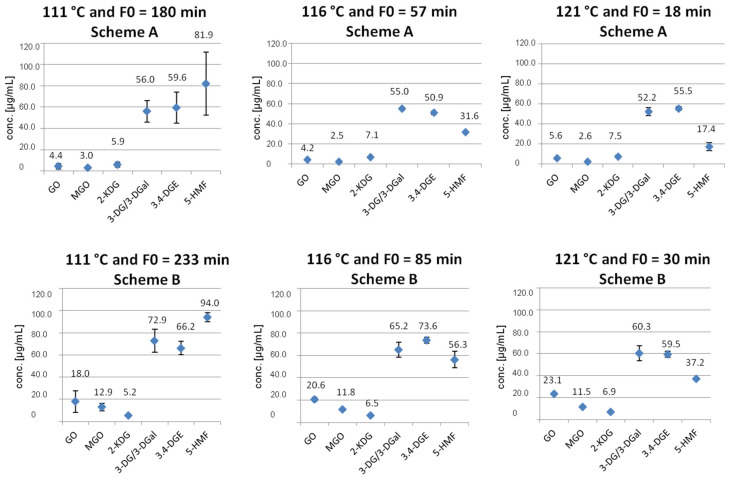
Comparison of the concentrations of GDPs formed at 111–116 °C autoclaved according to scheme A and according to scheme B (*n* = 27).

The 10% (*w*/*v*) glucose solutions autoclaved according to scheme B also show the same trend as described above: the concentrations of GO and 2-KDG increase with increasing temperature, whereas the concentrations of MGO, 3-DG/3-DGal, 3,4-DGE and especially 5-HMF decrease with increasing temperature. These results are shown in Figure 2 and Figure 3.

Figure 2 shows the different concentrations of GDPs formed at the respective temperatures according to scheme A versus scheme B. In comparison, it can be seen that higher concentrations of GDPs are formed in scheme B. The highest concentrations could be observed for the GDPs 3-DG/3-DGal and 5-HMF. The following Figure 3 shows an alternative representation.

Further figures showing the different concentrations of GDPs at the selected temperatures 111 °C, 116 °C, and 121 °C (schemes A and B) can be found in the Supplementary Materials (Figures S2–S13).

When the 10% (*w*/*v*) glucose solution is autoclaved at the standard autoclaving temperature of 121 °C for a much longer time (F0 = 202 min versus F0 = 18 min), thus exposing it to the high energy level for a much longer time, it can be observed that compared to scheme A, the concentrations of MGO, 2-KDG, 3-DG/3-DGal, and 3,4-DGE decrease by up to 85.3%, but the concentrations of GO and 5-HMF increase by up to 136.2% (Table 5).

In Table 5 it can be clearly seen that there are significantly lower concentrations of MGO, 2-KDG, 3-DG/3-DGal, and 3,4-DGE compared to the 10% glucose solution prepared at the standard autoclaving time (121 °C and F0 = 18 min). Table 5 is also shown in the Supplementary Materials (Figure S14).

The non-autoclaved 10% (*w*/*v*) glucose solution served as a reference value. Small amounts of GDPs (especially GO and MGO) were also observed (Table 6).

### 2.4. Content of GDPs in Commercially Available Aqueous Glucose Solutions (5–50%) in Different Types of Vessels from Three Different Manufacturers

In the following part of the experiment, different high concentrations of glucose solutions from three manufacturers A–C were investigated with regard to the occurrence and concentrations of GDPs. The glucose solutions had been autoclaved by the manufacturers A–C according to scheme A (F0 value of 18 min at 121 °C). Three measurements per batch were determined (Table 7, Table 8 and Table 9).

Comparing manufacturers A and B, it is noticeable that manufacturer A contains more GO and manufacturer B contains more 5-HMF in all glucose solutions (5–50%). Manufacturer C also has a comparatively very high proportion of GO and 5-HMF in relation to the 50% solution compared with manufacturers A and B.

With reference to the characteristics of the vessels of the marketed glucose solutions, it can be observed that in glass containers there are lower concentrations of GO and 2-KDG compared to PP bottles (50% glass versus 40% PP), despite the higher concentration of the glucose solutions. In return, there is a significant increase in 3-DG/3-DGal in the 50% glucose solutions autoclaved in glass vessels.

### 2.5. Method Validation via LC-MS/MS

The optimized method was validated according to the ICH Q2(R1) guideline [22].

#### 2.5.1. Selectivity

The LC-MS/MS method developed is selective for the GDPs investigated in this study. The glucose matrix did not affect the AUCs of the derivatized GDPs. Glucose was not derivatized at all and its presence did not affect the quantitative analysis of the GDPs. However, a limitation in terms of selectivity is that the method does not adequately separate 3-DG from 3-DGal.

#### 2.5.2. Linearity

All analytes could be well analyzed and evaluated and showed linear regression.

The 5-HMF concentration-dependent curve exhibited good correlation with $R^{2}=0.993$. 3,4-DGE exhibited very good correlation with $R^{2}=0.998$. GO, MGO, 2-KDG and 3-DG/3-DGal had excellent correlation coefficients with $R^{2}=0.999$. 3,4-DGE and 3-DG/3-DGal and 5-HMF were weighted $\frac{1}{x}$. Other GDPs were not weighted.

#### 2.5.3. Range

All GDP derivatives GO, MGO, 2-KDG, 3-DG/3-DGal, 3,4-DGE and 5-HMF provided adequate regression levels in the tested interval 0.5–100 µg/mL. The range was calculated as a compromise including all expected GDP concentrations.

#### 2.5.4. LOD

All derivatized GDPs had a LOD between 0.004 µg/mL and 0.078 µg/mL:


(1) LOD=3.3∗standard deviation of the response/slope of the calibration curve.

#### 2.5.5. LOQ

All derivatized GDPs had a LOQ between 0.012 µg/mL and 0.236 µg/mL:


(2) LOQ=10∗standard deviation of the response/slope of the calibration curve.

#### 2.5.6. Accuracy

Accuracy was reported as % recovery and tested in order to exclude possible systematic errors. The mean recovery (in%) was performed at three concentrations with six replicates each for the concentration levels 0.5, 25, and 100 µg/mL after a 16 h derivatization period with 0.75 mg/mL OPD. It ranged from 89.8 to 109.0%.

#### 2.5.7. Precision

The intraday precision was evaluated by analyzing three different concentrations with three replicates of each concentration. Intraday precision was calculated as RSD% for peak area. It ranged from 0.7 to 2.5% for GO, 0.7 to 1.4% for MGO, 0.5 to 2.9% for 2-KDG, 0.7 to 4.9% for 3-DG/3-DGal, 1.1 to 4.3% for 3,4-DGE and 1.0 to 3.4% for 5-HMF. All values of the RSD% are below 5%.

The validation results show that the method described here is a precise and reliable method for the quantification of GDPs in glucose solutions in the range indicated. All parameters of the method validation are presented in Table 10 and Table 11.

### 2.6. Statistical Analysis

Finally, a statistical analysis of the differences in concentrations of the individual GDPs autoclaved according to schemes A and B was carried out to evaluate the significance of the measured values (Table 12). The concentrations of the GDPs were compared per temperature and per GDP after they had been autoclaved either via scheme A or via scheme B.

A two sample *t*-tests was used to investigate if there was a significant difference between the two selected autoclaving schemes A and B (Table 1). Here, the content of each GDP per temperature of scheme A was compared with the content of each GDP within scheme B.

Subsequently, a two-sample t-test was performed based on the standard temperature of 121 °C, which was compared against the two alternative temperatures 111 °C and 116 °C with respect to the concentrations that occurred according to autoclaving scheme A (Table 13).

The two-sample t-test was performed in order to investigate if there is a significant difference between the standard autoclaving temperature 121 °C and the two alternative autoclaving temperatures, 111 °C and 116 °C, within scheme A. A clear significance can be seen for the GDPs 3,4-DGE (121 °C versus 116 °C) and 5-HMF (121 °C versus 111 °C and 121 °C versus 116 °C). At 121 °C, significantly lower concentrations of these GDPs are formed. This is in line with the graphical representation from Figure 2 and Figure 3.

## 3. Discussion

Known factors that can influence the content of GDPs are glucose concentration [7,17], pH [7], the chosen container [1,23,24], storage conditions [7], temperature [7], and heating time [12,13,14]. The last two influencing factors were examined in detail in this paper as they seem to be important components. A number of studies have already been carried out on different degradation products that have occurred in marketed medicinal products as well as in self-autoclaved glucose solutions after heat sterilization [12,13,14].

The objective of this work was to investigate the effect of different temperatures and autoclaving times on 10% (*w*/*v*) glucose solutions with regard to the formation of the six α-dicarbonyls GO, MGO, 2-KDG, 3-DG/3-DGal and 3,4-DGE as well as the aldehyde 5-HMF that occur after heat sterilization. In addition, suitable conditions to produce the lowest possible GDP concentrations were analyzed.

Due to possibly different activation energies and degradation kinetics of the various degradation reactions, the type and amount of GDPs obtained differ. The F0 value is derived on the basis of a first-order reaction and offers the possibility of comparing different heat treatment processes with one another or setting limit values for them [25,26,27]. The sterilization value is often used to optimize heat treatment processes, since most heat-related chemical degradation or formation reactions and predominantly also the killing of microorganisms can be described with the first-order reaction [25,26,27]. The killing of microorganisms takes place during heating phase, holding time, and cooling phase. The holding time is the variable part of heat sterilization. By autoclaving at higher temperatures, the required F0 value is achieved with a shorter total time. The holding time of the sterilization process can thus be shortened.

In Table 1, the calculated autoclaving times of the autoclaving schemes A and B were presented. The duration of the autoclaving procedure according to autoclaving scheme A was calculated according to the described formulae from the European Pharmacopoeia [4]. The equation used with its respective parameters is described in Section 4.2.2. In autoclaving scheme B, the calculated F0 time of scheme A was set as the holding time in the autoclave. Since the heating and cooling phases were added to the holding time, the autoclaving times for scheme B were significantly longer than for scheme A. Scheme B was used to investigate whether exceeding the recommended autoclaving time has a significant influence on the concentration of the resulting degradation products. The bioindicator *Geobacillus stearothermophilus* was used to check the effectiveness of killing all spores during the autoclaving process [28]. The successful killing of the germ during the autoclaving process was shown in Figure S1 (Supplementary Materials). Subsequently, the GDP concentrations obtained from the 10% glucose solutions autoclaved according to scheme A were compared with those obtained from scheme B for the temperatures 111 °C, 116 °C, and 121 °C. In addition, marketed 10% glucose solutions from different manufacturers A–C were analyzed and a validation of the LC-MS/MS method was carried out.

The glucose solutions exposed to scheme A with the shorter autoclaving times show lower concentration of GDPs (except for 2-KDG) than the glucose solutions autoclaved according to scheme B (longer autoclaving times). 2-KDG is apparently formed more preferentially at higher temperatures than at lower temperatures. However, in comparison from the longer to the shorter autoclaving time, it is more likely to be degraded with longer heat sterilization than with shorter autoclaving time. This was to be expected, as glucose degrades by oxidation, hydrolysis and dehydration, as shown in Figure 1. The longer moist heat is applied to glucose, the greater the proportion of GDPs formed. Comparing the concentrations of the GDPs at increasing temperatures within scheme A and scheme B, respectively, it is noticeable that the concentrations of GO and 2-KDG increase slightly with increasing temperature, whereas the concentrations of MGO, 3-DG/3-DGal, 3,4-DGE (exception 116 °C) and 5-HMF decrease.

Haybrard et al. describe glucose being degraded to 3-DG (and presumably also to its diastereomer 3-DGal) by enolization and dehydration [1]. From this, in turn, the α -DC MGO can be formed by breaking of bond (cleavage). Cyclisation of 3-DG (and 3-DGal) also produces 3,4-DGE. 3,4-DGE dehydrates further to 5-HMF. GO is an intermediate reactant, which is formed directly from glucose. This reaction sequence suggests that at higher temperatures and shorter autoclaving times, as shown in scheme A, slightly more intermediate reactants such as GO are formed from glucose, but all degradation products that depend on the intermediate 3-DG/3-DGal decrease. This means that enolization and dehydration of glucose decrease with increasing temperature and shorter autoclaving time.

In general, the concentrations of the GDPs 3-DG/3-DGal, 3,4-DGE, and 5-HMF (scheme A: 17.4–81.9 µg/mL; scheme B: 37.2–94.0 µg/mL) are many times higher than those of GO, MGO and 2-KDG (scheme A: 2.5–7.5 µg/mL; scheme B: 5.2–23.1 µg/mL), which is in line with the description of Haybrard et al. [1]. This means that the enolization and dehydration of glucose to 3-DG/3-DGal, its subsequent cyclization to 3,4-DGE and the subsequent dehydration to 5-HMF take place preferentially because they are energetically favored compared to the formation of GO, MGO and 2-KDG.

The standard autoclaving time of 121 °C and F0 = 18 min appears to produce the lowest levels of 3-DG/3-DGal and 5-HMF, but the highest levels of GO and 2-KDG.

Almost all GDPs (except 2-KDG) show lower concentrations in scheme A (=shorter autoclaving time). While GO, MGO, and 3,4-DGE show the significantly lowest concentrations in scheme A at 116 °C, the concentrations of 3-DG/3-DGal as well as of 5-HMF are lowest at 121 °C in scheme A. 2-KDG achieved the lowest values in scheme B at 111 °C (Table 4). Furthermore, the concentration of 5-HMF decreases with increasing temperature.

Tao et al. assumed that this is due to the hydrolytic degradation of 5-HMF to levulinic acid and formic acid [29]. Mannermaa et al. observed that the same applies to Ringer solutions: the use of the shortest sterilization cycle leads to the lowest 5-HMF concentrations in Ringer glucose solutions. Additional studies by Mannermaa et al. showed that at the same F0 value, the concentration of 5-HMF decreases the most at the highest temperatures [12,13,14]. Sturgeon et al. analyzed the breakdown of 10% dextrose solutions under simulated sterilization conditions. They investigated the autoclaved solutions at 102–132 °C and found that at all temperatures of heating, the formation rates of 5-HMF gradually increased with heating time [30]. We can confirm this finding with our results. Comparing the concentrations for 5-HMF within the schemes A and B, respectively, we found that less 5-HMF was formed at higher temperatures where heating was shorter compared to colder autoclaving temperatures where autoclaving was longer (e.g., 121 °C versus 111 °C).

When comparing the self-autoclaved glucose solutions according to scheme A (121 °C, F0 = 18 min) with the industrially produced counterpart of manufacturers A, B, and C, it is noticeable that generally for manufacturers A–C the values for GO are clearly higher than our determined values. For MGO, 2-KDG, 3-DG/3-DGal, 3,4-DGE, and 5-HMF, the 10% (*w*/*v*) glucose solutions of manufacturers A–C showed significantly lower contents, regardless of the nature of the vessels. Looking at the 5–50% (*w*/*v*) glucose solutions from manufacturers A–C, we see that all manufacturers have the highest GDP values for GO and 3-DG/3-DGal (Table 7, Table 8 and Table 9). MGO has the lowest concentrations in all solutions. Compared to our autoclaved solutions corresponding to scheme A, it is noticeable that we obtain much lower values of GO in the 10% (*w*/*v*) glucose solution, but slightly higher values for MGO, 2-KDG, and significantly higher values for 3-DG/3-DGal, 3,4-DGE, and 5-HMF. This may be due to the different autoclaving devices or possibly also additives that the manufacturers used to adjust the pH of their glucose solutions. In accordance with the national monograph, we did not use such additives here. The rate-determining step in the formation of GDPs is not solely dependent on the respective glucose concentration and this process is not based on a linear reaction mechanism. This can be seen from the fact that, for example, the 20% glucose solutions from manufacturers A–C do not have twice the content of GDPs compared to the 10% glucose solution.

The examination of the glucose solutions autoclaved at the standard temperature of 121 °C for 350 min (F0 = 202 min) with a significantly longer heat exposure, showed an increase in the concentrations of GO and 5-HMF and a decrease in the concentrations of MGO, 2-KDG, 3-DG/3-DGal, and 3,4-DGE (Table 5). This means that the formation of GO and 5-HMF is favored with long heat exposure, while the other degradation products decompose.

Regarding the pH values, it can be concluded that the non-autoclaved 10% (*w*/*v*) glucose solution measured at room temperature with pH = 4.976 has a significantly more acidic pH value than double distilled water (pH = 6.812). A clear trend was seen that with increasing temperature and also with increasing autoclaving time, the pH becomes more acidic. Since GDPs have an acidic pH [31,32], it is reasonable to assume that many acidic GDPs are produced due to the influence of temperature and autoclaving time. The comparison of autoclaved double-distilled water also showed this trend, which is due to the fact that CO_2_ from the air is absorbed and bound by the double-distilled water and carbonic acid is formed. Mannermaa et al. found, that the pH of the solutions decreases during sterilization, with the exception of F0 values at 5–15 min [12]. With 20% glucose solutions, stored at room temperature for 30 days, the pH value decreases by approx. 0.20 units [12]. This is also the same in our study. A possible explanation might be that more GDPs are formed, which are acidic by their nature. This phenomenon is also observed with longer storage times [1]. Haybrard et al. have conducted an analysis of covariance (ANCOVA). They showed that there is a significant influence of storage time and oxygen permeability on the formation rates of 5-HMF in sterile glucose solutions for infusion [1]. This is in line with observations from Kjellstrand et al. who have reported that the most important factor determining the rate of GDP production during storage was temperature [7]. The GDPs created by heat sterilization promoted further degradation. They stated, that at a storage temperature of 20 °C and a pH of 3.2, degradation was almost negligible. They found that after 2 years at 40 °C, the concentrations of GDPs produced during storage were of the same magnitude as those caused by heat sterilization.

The validation of the method presented in this work confirms that the investigated parameters were all within the required ranges of the ICH guideline. Compared with the work of Mittelmaier et al. [8], it can be stated that in the present study somewhat higher values for LOD and LOQ were determined for the GDPs GO, MGO, 2-KDG (LOD: 0.30–1.34 µM, LOQ: 0.90–4.07 µM). When investigating marketed single- and double-chamber peritoneal dialysis (PD) fluids, Mittelmaier et al. [8] achieved values for LOD from 0.13 to 0.19 μM and for LOQ from 0.40 to 0.57 μM. However, more sensitive detection and quantification values were obtained for 3-DG/3-DGal as well as for 3,4-DGE and 5-HMF (LOD: 0.02–0.10 µM and LOQ 0.07–0.32 µM).

A useful factor to add to the assessment of the ideal autoclave condition is the fact of toxicity. There are many divergent studies on 5-HMF, which examined the possible genotoxic or carcinogenic potential of 5-HMF [33]. Janzowski et al. found that 5-HMF induced moderate cytotoxicity. DNA damage was not measurable. 5-HMF was weakly mutagenic at concentrations between 80 and 140 mM [34]. This corresponds to a concentration of 10.1–17.7 mg/mL. Ulbricht et al. found that very high levels of 5-HMF exceeding 75 mg/kg body weight may lead to acute toxicity [35].

According to the German national monograph of glucose solutions for parenteral use, however, the limits for 5-HMF are significantly lower, with a maximum of 44 µg/mL. Thus, the possible toxicity level is not reached by far. Although the toxic potential of 5-HMF has been much discussed in the past, it has been classified as not harmful to health according to the safety data sheet and by the German Federal Institute for Risk Assessment (BfR) [36].

After classification of the seven investigated degradation products according to EU Chemicals Regulation (EC) No 1272/2008 [37] it can be stated that especially GO and MGO were classified as potentially germ cell mutagenic (category 2, H341) and that sensitization by skin contact (category 1, H317) is possible [18,19]. In addition, for 3,4-DGE there is a risk of skin corrosion/irritation (category 2) as well as for serious eye damage/eye irritation (category 2) [38]. For 5-HMF there is a potential irritant effect on the skin (category 2), H315 as well as for eye irritation (category 2), H319 [39]. 2-KDG, 3DG, and 3-DGal are not evaluated as hazardous substances or mixtures according to Directive (EC) No 1272/2008 [40,41,42]. Since a health risk may arise in particular from the GDPs GO and MGO [18,19], it would be useful to set limits here for the presence of these GDPs in glucose infusion solutions.

However, we already found approximately 1 µg/mL for GO and MGO in the non-autoclaved 10% glucose solution that served as reference solution. The fact that a small amount of glucose is enolized and dehydrated even without heat sterilization (Table 6) should be further observed due to the different information on toxicity.

Disadvantages for the human health status result mainly from the further reaction of GDPs in the human body, as it is known that GDPs are highly reactive molecules that bind to serum proteins and lead to the formation of advanced glycation end products (AGEs) [1,6,43]. AGEs increase the oxidative stress of cells [44,45] and accumulate in vessels [11]. They affect the cardiovascular system [44,46,47,48] and are associated with an increase in cardiovascular morbidity [49] and strokes [50]. They also play a causative role in vascular complications of diabetes mellitus [51], Alzheimer’s disease [52], and deterioration of kidney function [5,10,53].

## 4. Materials and Methods

### 4.1. Reagents and Chemicals

For all experiments, freshly prepared ultrapure water was taken from Sartorius arium^®^ pro UV water treatment system (Sartorius AG, Göttingen, Germany). All chemicals were of analytical grade, unless noted otherwise. Acetonitrile, OPD, 2,3-dimethylquinoxaline, MGO, GO, 2-KDG, 3-DG, 5-HMF, D-(+)-glucose monohydrate, methanol and ammonium acetate were purchased from Sigma (Sigma-Aldrich Chemie GmbH, Steinheim, Germany). 3-DGal was obtained from Cayman (Cayman Chemical Company, Ann Arbor, MI, USA). 3,4-DGE was purchased from Carbosynth (Carbosynth Ltd., Compton, Berkshire, UK).

The aqueous phase during LC-MS/MS measurement was a 5 mM ammonium acetate buffer solution adjusted to pH = 3.5 using 0.1% (*v*/*v*) acetic acid. It was freshly prepared in accordance to Thomas et al. [54]. The EZ-Test^®^ [55] Biological Indicator from MesaLabs was used in order to test sterility.

### 4.2. Experimental Overview

In order to investigate the effects of heat and exposure time on heat sterilized glucose solutions, which can lead to different concentrations of GDPs formed, 10% (*w*/*v*) glucose solutions were prepared and heat sterilized according to the F0 concept, which describes the sum of all lethal effects acting on a population of the key germ *Geobacillus stearothermophilus* in the course of heating. Furthermore, the identity and amount of these GDPs were analyzed using a slightly modified LC-MS/MS method described by Mittelmaier [8]. The autoclaving process was performed in polypropylene (PP) bottles that were heat resistant up to 121 °C. The amounts of GDPs formed in the different temperature/time constellations were determined and compared, as were the pH values of the autoclaved and non-autoclaved solutions. In addition, 10% (*w*/*v*) aqueous glucose solutions in PP bottles from a finished drug manufacturer were tested for the presence of GDPs and compared to the extent of GDPs formed from the self-autoclaved glucose solutions at 121 °C.

#### 4.2.1. Preparation of Glucose Solutions

A total of 11 L of a 10% (*w*/*v*) glucose solution were prepared according to the German standard approval monograph [20]. Double distilled water and glucose monohydrate were used for this purpose. The solution was then sterile filtered through pre-sterilized Stericup and Steritop^®^ Vaccuum Driven Disposable Filtration System with 0.22 µm filter membranes (Merck Milipore Express PLUS). Filtration was performed directly into the final containers, which were sterilized by autoclaving. The final containers to be sterilized were 225 mL PP bottles (Kautex™), each filled with 200 mL glucose solution.

#### 4.2.2. Calculation of the Required Steam Sterilization Time

The reference cycle for steam sterilization is 15 min at 121 °C in saturated steam, with the temperature measured at the coldest point of the chamber [2]. The calculation of the sterilization efficiency with the F0 concept was performed by the following equation

(3) $$F0=\left(\log\;N0-\log\;Nt\right)\;*\;\left(D2\;*\;10^{\frac{\left(T2-T1\right)}{z}}\right),$$

which is described in the general text of the European Pharmacopoeia [4].

N0 is the assumed initial germ load of 10^6^. N*t* is the target final germ load after the autoclaving process 10^−6^. The D2 value of the reference germ *Bacillus stearothermophilus* at 121 °C is 1.5. The D-value (or decimal reduction value) is the value of a sterilization parameter (duration or absorbed dose) required to reduce the number of reproducible units to 10 percent of the initial value. T2 is the standard temperature of 121 °C. T1 is the selected temperature (111 °C; 116 °C) and z is the temperature change necessary to change the D value by a factor of 10. The value 10 was assumed for z.

The F0 value thus obtained is now the new autoclaving time at the selected temperature and for the corresponding germ (= overkill condition). F0 can be described most simply as the equivalent time required in minutes at 121 °C to produce the same microbiological killing effect as the process used [56].

The calculated F0 values for the overkill procedure according to scheme A were shown in Table 1. The total F0 value of a process (unit: minutes) takes into account the heating and cooling phases of the cycle. The 10% (*w*/*v*) glucose solutions calculated and autoclaved in accordance with the F0 concept (scheme A) were also to be compared with solutions that were specifically autoclaved for too long (scheme B), in order to investigate not only the influence of the temperature but also that of the exposure time.

For the calculations of the F0 times of scheme B, the F0 times for heating up and cooling down the glucose solutions were added to the F0 times from A. This is also shown in Table 1. Three batches per selected temperature from schemes A and B were autoclaved, each batch consisting of three bottles, which were processed after cooling. In addition, three batches with three bottles (*n* = 9) of 10% (*w*/*v*) glucose solution were heat sterilized at 121 °C for 350 min (F0 = 202 min). The purpose of this experiment was to investigate an extreme situation in terms of autoclaving time with respect to the concentrations of GDPs occurring at the standard temperature of 121°C. Three bottles of a non-autoclaved 10% (*w*/*v*) glucose solution were also analyzed (reference value).

#### 4.2.3. Autoclaving under Germicidal Control

The Varioklav EC from Thermo Scientific was used for heat sterilization. The 10% (*w*/*v*) glucose solutions in PP bottles were autoclaved at 111 °C, 116 °C, and 121 °C in their final container. To each autoclave run, an ampoule of EZ-Test^®^ from Mesa Labs containing the heat sterilization lead germ *Geobacillus stearothermophilus*, culture 7953, at a concentration of approximately 1 × 10^5^ to 1 × 10^6^ was added to check for successful bacterial kill. In addition to the germ *Geobacillus stearothermophilus*, the ampoule EZ-Test^®^ contains a nutrient solution based on soybean casein digest, as well as the violet pH indicator bromocresol purple. The ampoule was placed in another container filled with water, which was analogous to the final container to be autoclaved (e.g., when autoclaving the PP bottles, the EZ-Test^®^ was placed in another PP bottle filled with water to mimic the conditions in the final container to be sterilized).

After the autoclaved EZ-Test^®^ ampoules had cooled in the fume hood for 10 min, the culture medium and the indicator were activated. For this, the ampoules were placed in an upright position and gently squeezed to break the glass ampoules by hand. The growth media was allowed to come in contact with the spores of *Geobacillus stearothermophilus*.

These ampoules were then placed in an incubator rack in a GFL 3032 incubator and incubated together with an unsterilized ampoule, which was also crushed. The ampoules were incubated at 60 °C for 24 h and afterwards observed for color change.

#### 4.2.4. Measuring of the pH Values

To evaluate the influence of the pH value on the formation of GDPs formed by heat sterilization, the pH value was determined in all autoclaved and non-autoclaved glucose solutions as well as in the control samples.

#### 4.2.5. Preparation of Calibration Solutions

A stock solution containing GO, MGO, 2-KDG, 3-DG, 3-DGal, 3,4-DGE, and 5-HMF, each with a concentration of 100 µg/mL per GDP, was prepared in bi-distilled water in amber vials. This stock solution was diluted with bi-distilled water to obtain a concentration of 2 µg/mL. From this solution, a dilution series was prepared ranging from 0.005 to 0.85 µg/mL. All solutions of the calibration series also contained 0.1 mg/mL glucose, as well as 0.75 mg/mL OPD and 5 µg/mL of the internal standard 2,3-dimethylquinoxaline.

#### 4.2.6. Derivatization of Autoclaved Glucose Solutions

The heat sterilized glucose solutions described in Section 4.2.1. and those from the manufacturers A–C were diluted with water in a ratio of 1:1000. The diluted solutions also contained 0.75 mg/mL OPD and 5 µg/mL of the internal standard (2,3-dimethylquinoxaline).

All solutions were left in the dark for 16 h and were subsequently analyzed via LC-MS/MS. The suitability of the chosen derivatization procedure in terms of OPD concentration and derivatization time has been shown elsewhere [57].

#### 4.2.7. LC-MS/MS Analysis

The development of an LC-MS/MS method was based on the process reported by Mittelmaier et al. [8], which we optimized. Qualitative analysis and structure elucidation was performed by LC-MS/MS. The respective LC-MS/MS parameters and ion transitions are shown in Table 14.

Liquid chromatography was performed on a Shimadzu Nexera ultra-fast liquid chromatograph (UFLC) equipped with an analytical C18 column (Nucleoshell RP 18, 100 mm × 3 mm, 2.7 µm particle size, Macherey-Nagel, Dueren, Germany). The UHPLC system (degasser, binary pump, autosampler, column oven) was coupled to a SCIEX QTrap6500 triple quadrupole mass spectrometer (Sciex, Darmstadt, Hessen, Germany) and operated under positive electrospray ionization (ESI) conditions with a needle voltage of 5500 V at 450 °C and nitrogen as drying gas.

The collision energies were 40 eV for the internal standard (2,3-Dimethylquinoxaline), MGO- and GO- and 20 eV for 2-KDG- and 3,4-DGE- and 25 eV for 3-DG-, 3-DGal- and 5-HMF-derivatives, respectively. Mobile phase A consisted of a 5 mM ammonium acetate buffer solution adjusted to pH 3.5 using 0.1% (*v*/*v*) acetic acid, and mobile phase B consisted of acetonitrile. The total flow rate was 0.35 mL/min. The gradient started at 5% solvent B, remained isocratic for 0.2 min, and increased to 50% B within 10 min. From 10.00 to 10.01 it increased to 100% B, remaining there for 1 min. The column was re-equilibrated from 11.01 min to 14.00 min at 5% B. The overall run time was 14 min. The injection volume was 5 μL. System control, data acquisition, and processing were performed by Analyst 1.6.2 software.

#### 4.2.8. Method Validation

To validate the method, all parameters listed in the ICH Q2 (R1) guideline [22] were considered: Accuracy (reported as percent recovery), precision, linearity, range, limit of detection (LOD), and limit of quantitation (LOQ) were determined.

In order to calculate the accuracy (reported as% recovery), an unheated 10% (*w*/*v*) glucose solution fluid was spiked with 0.5, 25, and 100 µg/mL of each GDP, 5 µg/mL internal standard (2,3-dimethylquinoxaline) and 0.75 mg/mL OPD (i.e., three concentrations/six replicates). These samples and an unspiked fluid were analyzed via LC-MS/MS as described in Section 4.2.7. after 16 h of derivatization. The mean recovery of three experiments for each concentration level was determined and expressed as: (GDP concentration-GDP concentration of the unspiked sample)/added GDP concentration×100% (Table 11). Precision was expressed as standard deviation and coefficients of variation (% RSD). Nine determinations covering the specified range for the procedure (three concentrations/three replicates each) were made. The mean value, the standard deviation, and the precision were calculated (Table 11). The LOD was expressed as (3.3 × standard deviation of the response)/slope of calibration curve and LOQ was expressed as (10 × standard deviation of the response)/slope of calibration curve [22]. LOD and LOQ are shown in Table 10. An eight-point-calibration curve in order to determine linearity was prepared in three replicates (0.5–100 µg/mL each GDP in water as well as 10% glucose, 5 µg/mL 2,3-dimethylquinoxaline and 0.75 mg/mL OPD). The calibration curve was obtained by plotting the quotient of the peak areas of the derivatized GDPs and the internal standard (ordinate) against the concentration of the derivatized GDPs (abscissa). Calibration lines are shown in Table 10. Linear regression analysis was used to assess the linearity of the calibration curve. Regression parameters were computed using Excel (Microsoft Office Professional Plus 2016).

#### 4.2.9. Statistical Analysis

The GDP concentrations that occurred at the three temperatures 111 °C, 116 °C, and 121 °C in scheme A should be compared with the analogously measured GDP concentrations of scheme B in order to test for differences in the mean GDP concentration.

In addition, the standard temperature of 121 °C was to be tested against the two temperatures 111 °C and 116 °C with respect to the differences in the measured GDP concentration within scheme A. For this purpose, we used the classical t-test analysis.

The degree of freedom for each of the two-sample tests was 16, since each group consisted of exactly 9 samples. To account for multiple testing, we calculated Bonferroni-corrected *p*-values in addition to the nominal values for each test. All tests were performed by the use of the statistic software environment R-4.0.2.

## 5. Conclusions

A previously described LC-MS/MS method for quantitative analysis of GDPs typically formed during heat sterilization of glucose solutions was slightly modified and validated according to the ICH Q2(R1) guideline [22]. The modified method was demonstrated to be precise, sensitive, and reproducible and thus suited to screen and simultaneously quantify the content of all 7 GDPs.

After analyzing marketed 10% glucose solutions from manufacturers A–C, as well as 10% glucose solutions prepared and autoclaved according to autoclaving schemes A/B, and taking into account factors such as toxicity, it may be appropriate to change the standard conditions from 121 °C and 15 min to 116 °C and F0 = 57 min. The main reason for this recommendation is that the lowest concentrations of the toxic GDPs GO and MGO occurred when the autoclaving temperature and duration were changed to 116 °C and F0 = 57 min. In this autoclaving scheme, the concentration of 5-HMF was 31.6 µg/mL, still well below the limit of 44 µg/mL required by the national monograph.

Another advantage for industry in the large-scale production of glucose solutions could also be that the proposed new autoclaving temperature of 116 °C is 5 °C below the temperature of the reference process, thus possibly saving energy costs. However, the autoclaving time would also be longer compared to 121 °C and 15 min. The actual energy costs would have to be determined in further trials.

## Figures and Tables

**Table 1:** Autoclaving schemes A/B.

Temperature [°C]	Scheme A (Overkill Conditions) Autoclaving Time (F0) [min]	Scheme B Autoclaving Time (F0) [min]
111	180	233
116	57	85
121	18	30

**Table 2:** Overview of the autoclaving results with regard to the germ *Geobacillus stearothermophilus*.

Temperature [°C]	F0 [min] (Scheme A)	*Geobacillus stearothermophilus* Killed (Scheme A/Scheme B)?
111	180	yes
116	57	yes
121	18	yes

**Table 3:** Overview of the obtained pH-values ± SD (= standard deviation) in different temperatures/sterilization-times/vessels.

	10% Glucose Solution (*n =* 3 for Each Scheme A/B)		Control Values of Autoclaved Water without Glucose (*n =* 3 for Each Scheme A/B)	
Temperature [°C]	10% PP Bottle (Scheme A) pH ± SD	10% PP Bottle (Scheme B) pH ± SD	Water, PP Bottle (Scheme A) pH ± SD	Water, PP Bottle(Scheme B) pH ± SD
111	5.17 ± 0.0	4.08 ± 0.0	7.03 ± 0.0	6.80 ± 0.0
116	4.67 ± 0.0	4.12 ± 0.0	6.77 ± 0.0	6.53 ± 0.0
121	4.36 ± 0.0	4.15 ± 0.0	6.68 ± 0.0	6.11 ± 0.0
non-autoclaved (room temperature)	4.98 ± 0.0		6.81 ± 0.0	

**Table 4:** Concentrations of GDPs in 10% (*w*/*v*) glucose solution in PP bottles autoclaved according to scheme A (*n =* 27).

	Temp [°C]	GO [µg/mL] ± SD	MGO [µg/mL] ± SD	2-KDG [µg/mL] ± SD	3-DG/3-DGal [µg/mL] ± SD	3,4-DGE [µg/mL] ± SD	5-HMF [µg/mL] ± SD
**Scheme A**	111	4.4 ± 2.7	3.0 ± 0.3	5.9 ± 2.2	56.0 ± 10.2	59.6 ± 14.5	81.9 ± 29.5
	116	4.2 ± 0.7	2.5 ± 0.2	7.1 ± 0.6	55.0 ± 1.6	50.9 ± 1.7	31.6 ± 0.5
	121	5.6 ± 1.3	2.6 ± 0.2	7.5 ± 1.4	52.2 ± 4.0	55.5 ± 1.7	17.4 ± 3.9
**Scheme B**	111	18.0 ± 9.7	12.9 ± 3.2	5.2 ± 0.3	72.9 ± 10.5	66.2 ± 6.2	94.0 ± 4.0
	116	20.6 ± 1.7	11.8 ± 0.4	6.5 ± 0.8	65.2 ± 6.7	73.6 ± 2.8	56.3 ± 7.4
	121	23.2 ± 0.9	11.5 ± 0.8	6.9 ± 0.7	60.3 ± 6.8	59.5 ± 2.8	37.2 ± 0.8

**Table 5:** Concentrations of GDPs in 10% (*w*/*v*) glucose solutions in PP bottles heat sterilized at 121 °C for 350 min (F0 = 202 min). 3 batches with 3 bottles each were analyzed (*n* = 9).

Temp [°C]	GO [µg/mL] ± SD	MGO [µg/mL] ± SD	2-KDG [µg/mL] ± SD	3-DG/3-DGal [µg/mL] ± SD	3,4-DGE [µg/mL] ± SD	5-HMF [µg/mL] ± SD
121	8.3 ± 0.0	1.2 ± 0.1	1.1 ± 0.1	13.7 ± 0.1	12.5 ± 0.3	41.1 ± 0.1

**Table 6:** Concentrations of GDPs in 10% (*w*/*v*) glucose solutions in PP bottles that were not heat sterilized (reference value). Three bottles were analyzed (*n* = 3).

Temp [°C]	GO [µg/mL] ± SD	MGO [µg/mL] ± SD	2-KDG [µg/mL] ± SD	3-DG/3-DGal [µg/mL] ± SD	3,4-DGE [µg/mL] ± SD	5-HMF [µg/mL] ± SD
121	1.0 ± 0.5	0.9 ± 0.1	0.1 ± 0.0	n.d.	n.d.	0.1 ± 0.0

n.d. = not detectable.

**Table 7:** Concentrations of GDPs. Manufacturer A (*n* = 3).

MAH/Conc	GO [µg/mL] ± SD	MGO [µg/mL] ± SD	2-KDG [µg/mL] ± SD	3-DG/3-DGal [µg/mL] ± SD	3,4-DGE [µg/mL] ± SD	5-HMF [µg/mL] ± SD
5% PP	35.9 ± 2.5	0.8 ± 0.3	0.5 ± 0.6	8.5 ± 0.7	1.2 ± 1.0	0.4 ± 0.6
10% PP	42.1 ± 8.5	0.8 ± 0.3	1.1 ± 0.9	11.3 ± 1.0	1.5 ± 1.3	0.7 ± 0.6
20% PP	39.2 ± 3.4	0.8 ± 0.2	18.9 ± 4.8	1.3 ± 0.1	0.1 ± 0.1	2.1 ± 0.2
40% PP	47.3 ± 2.6	0.9 ± 0.2	23.0 ± 0.3	0.4 ± 0.1	0.0 ± 0.0	4.6 ± 0.4
50% Glass	43.6 ± 3.9	0.9 ± 0.4	11.1 ± 1.0	31.4 ± 2.4	3.5 ± 0.3	5.6 ± 0.9

**Table 8:** Concentrations of GDPs. Manufacturer B (*n* = 3).

MAH/Conc	GO [µg/mL] ± SD	MGO [µg/mL] ± SD	2-KDG [µg/mL] ± SD	3-DG/3-DGal [µg/mL] ± SD	3,4-DGE [µg/mL] ± SD	5-HMF [µg/mL] ± SD
5% PP	0.6 ± 0.1	0.5 ± 0.1	1 ± 0.9	14.2 ± 3.4	3.4 ± 1.0	1.6 ± 0.3
10% PP	11.1 ± 0.5	0.6 ± 0.1	1.7 ± 0.1	18.4 ± 2.1	3.6 ± 0.4	2.6 ± 0.4
20% PP	13.7 ± 1.3	0.7 ± 0.3	5.1 ± 2.7	20.2 ± 0.4	3.3 ± 0.3	5.0 ± 0.3
40% PP	16.1 ± 3.6	0.8 ± 0.5	17.5 ± 8.8	20.4 ± 0.4	1.6 ± 0.0	9.5 ± 1.2
50% Glass	15.9 ± 0.9	0.9 ± 0.2	10.1 ± 2.6	34.6 ± 0.5	4.0 ± 0.3	7.9 ± 1.0

**Table 9:** Concentrations of GDPs. Manufacturer C (*n* = 3).

MAH/Conc	GO [µg/mL] ± SD	MGO [µg/mL] ± SD	2-KDG [µg/mL] ± SD	3-DG/3-DGal [µg/mL] ± SD	3,4-DGE [µg/mL] ± SD	5-HMF [µg/mL] ± SD
5% Glass	20.9 ± 5.1	0.7 ± 0.3	0.3 ± 0.6	12.3 ± 0.7	2.9 ± 0.2	1.2 ± 0.1
10% Glass	31.9 ± 3.2	0.7 ± 0.2	0.9 ± 0.9	13.3 ± 1.4	2.5 ± 0.2	2.9 ± 0.2
20% Glass	33.0 ± 0.8	0.7 ± 0.1	7.6 ± 4.2	14.2 ± 0.6	1.2 ± 0.2	14.1 ± 1.1
40% PP	32.4 ± 6.5	0.8 ± 0.4	15.3 ± 2.3	10.8 ± 1.1	0.8 ± 0.1	4.6 ± 0.1
50% Glass	40.5 ± 3.3	0.9 ± 0.3	6.7 ± 4.5	31.8 ± 3.0	2.9 ± 0.2	12.9 ± 0.5

**Table 10:** Method validation parameters.

**Analyte**	**Regression**	**R^2^**	Weighting	Range [µg/mL]	LOD [µg/mL] (^1^ calc.)	LOQ [µg/mL] (^1^ calc.)
GO	$\;y=1.01040x+4.55224e^{-4}$	0.999	none	0.5–100	0.078	0.236
MGO	y=3.30991x+0.00154	0.999	none	0.5–100	0.023	0.070
2-KDG	$y=1.98896x-2.01261e^{-5}$	0.999	none	0.5–100	0.053	0.161
3-DG/3-DGal	y=13.02104x+0.00448	0.999	$\frac{1}{x}$	0.5–100	0.004	0.012
3,4-DGE	$y=5.15815x+5.56583e^{-4}$	0.998	$\frac{1}{x}$	0.5–100	0.015	0.046
5-HMF	y=11.23859x+0.01012	0.993	$\frac{1}{x}$	0.5–100	0.010	0.031

^1^ calc. = calculated according to ICH Q2(R1) guideline [22].

**Table 11:** Precision in terms of % relative standard deviation (RSD) for replicate measurements (*n* = 3) at three different levels, and accuracy reported as percent recovery for three concentrations/six replicates each of the total analytical procedure.

		Precision (as% RSD)		Accuracy (% Recovery)	
GDP	GDP conc. [µg/mL]	Mean [µg/mL] ± SD	RSD%	Mean [µg/mL] ± SD	% Recovery
GO	0.5	0.5 ± 0.0	2.5	0.5 ± 0.0	98.8
	25	23.0 ± 0.3	1.2	24.6 ± 0.7	98.3
	100	98.5 ± 0.7	0.7	98.2 ± 3.0	98.2
MGO	0.5	0.7 ± 0.0	0.8	0.5 ± 0.0	109.0
	25	24.0 ± 0.3	1.4	25.5 ± 0.7	102.1
	100	98.5 ± 0.7	0.7	99.5 ± 2.4	99.5
2-KDG	0.5	0.6 ± 0.0	2.9	0.5 ± 0.0	103.7
	25	24.7 ± 0.6	2.4	26.0 ± 0.9	103.9
	100	101.5 ± 0.5	0.5	103.6 ± 3.7	103.6
3-DG/ 3-DGal	0.5	0.4 ± 0.0	4.9	0.4 ± 0.0	89.8
	25	26.4 ± 0.2	0.7	25.1 ± 1.8	100.2
	100	99.3 ± 2.0	2.0	100.5 ± 3.1	100.5
3,4-DGE	0.5	0.5 ± 0.0	4.3	0.5 ± 0.0	106.9
	25	25.9 ± 0.5	2.0	26.0 ± 2.0	104.2
	100	92.3 ± 1.1	1.1	96.1 ± 4.0	96.1
5-HMF	0.5	0.5 ± 0.0	3.1	0.5 ± 0.0	94.8
	25	26.9 ± 0.3	1.4	27.0 ± 0.7	107.9
	100	103.4 ± 3.6	0.5	102.5 ± 4.5	102.5

**Table 12:** Comparison of the two autoclaving schemes A and B with regard to the influence of temperature on the resulting GDP concentrations.

GDP Temp [°C]		F0 Scheme A vs. F0 Scheme B		
		*p*-Value	Significance Level	Degrees of Freedom
GO	111 °C	0.008012	*	16
	116 °C	1.512 × 10^−12^	*/**	
	121 °C	1.516 × 10^−13^	*/**	
MGO	111 °C	0.3065	not significant	
	116 °C	6.058 × 10^−14^	*/**	
	121 °C	2.938 × 10^−13^	*/**	
2-KDG	111 °C	0.5692	not significant	
	116 °C	0.1991	not significant	
	121 °C	0.4838	not significant	
3-DG/ 3-DGal	111 °C	0.05203	not significant	
	116 °C	0.003162	*	
	121 °C	0.01666	*	
3,4-DGE	111 °C	0.4311	not significant	
	116 °C	6.311 × 10^−8^	*/**	
	121 °C	0.02456	*	
5-HMF	111 °C	0.3691	not significant	
	116 °C	1.03 × 10^−6^	*/**	
	121 °C	4.946 × 10^−9^	*/**	

* p ≤ 0.05: significant on nominal significance level ** p ≤ 0.003 (0.05/18): significant after Bonferroni correction for multiple testing.

**Table 13:** Comparison of GDP concentrations at 121 °C versus the two alternative autoclaving temperatures 116 °C and 121 °C in autoclaving scheme A.

GDP	121 °C versus 111 °C and 116 °C				
	Standard Autoclaving Temperature	Alternative Autoclaving Temperature	*p*-Value	Significance Level	Degrees of Freedom
GO	121 °C	111 °C	0.3051	not significant	16
	121 °C	116 °C	0.05448	not significant	
MGO	121 °C	111 °C	0.1987	not significant	
	121 °C	116 °C	0.6584	not significant	
2-KDG	121 °C	111 °C	0.2506	not significant	
	121 °C	116 °C	0.6102	not significant	
3-DG/ 3-DGal	121 °C	111 °C	0.6078	not significant	
	121 °C	116 °C	0.1801	not significant	
3,4-DGE	121 °C	111 °C	0.6067	not significant	
	121 °C	116 °C	0.01357	*	
5-HMF	121 °C	111 °C	1.256 × 10^−4^	*/**	
	121 °C	116 °C	5.134 × 10^−7^	*/**	

* p ≤ 0.05: significant on nominal significance level ** p ≤ 0.004 (0.05/12): significant after Bonferroni correction for multiple testing.

**Table 14:** Parameters of the LC-MS/MS analysis.

ID	Q1 [*m/z*]	Q3 [*m/z*]	Dwell Time [msec]	CE [eV]	DP [eV]	R_t_ [min]
GO	131.1	76.7	50	40	100	6.95
MGO	145.1	77	50	40	100	7.80
2-KDG	251.1	173.2	50	20	100	3.51
3-DG/3-DGal	235.1	199.1	50	25	100	4.79
3.4-DGE	217.1	169.1	50	20	100	6.37
5-HMF	215.1	197.1	50	25	100	6.46
IS	159.1	118.1	50	40	100	8.48

ID = Identity of analyte, Q1 = Quadrupole 1, Q3 = Quadrupole 3, CE *=* Collision energy, DP = Declustering Potential, R_t_ = Retention time.

## Data Availability

All data are contained within the article.

## Supplementary Materials

The following are available online at https://www.mdpi.com/article/10.3390/ph14111121/s1, Figure S1: EZ-Test^®^ for the control of germ killing of the lead germ Geobacillus stearothermophilus after heat sterilization, Figure S2: Concentrations of GO after heat sterilization of 10% glucose solutions at 111 °C, 116 °C, and 121 °C autoclaved according to scheme A (*n* = 27), Figure S3: Concentrations of MGO after heat sterilization of 10% glucose solutions at 111 °C, 116 °C, and 121 °C autoclaved according to scheme A (*n* = 27)*,* Figure S4: Concentrations of 2-KDG after heat sterilization of 10% glucose solutions at 111 °C, 116 °C, and 121 °C autoclaved according to scheme A (*n* = 27), Figure S5: Concentrations of 3-DG/3-DGal after heat sterilization of 10% glucose solutions at 111 °C, 116 °C, and 121 °C autoclaved according to scheme A (*n* = 27), Figure S6: Concentrations of 3,4-DGE after heat sterilization of 10% glucose solutions at 111 °C, 116 °C, and 121 °C autoclaved according to scheme A (*n* = 27), Figure S7: Concentrations of 5-HMF after heat sterilization of 10% glucose solutions at 111 °C, 116 °C, and 121 °C autoclaved according to scheme A (*n* = 27), Figure S8: Concentrations of GO after heat sterilization of 10% glucose solutions at 111 °C, 116 °C, and 121 °C autoclaved according to scheme B (*n* = 27), Figure S9: Concentrations of MGO after heat sterilization of 10% glucose solutions at 111 °C, 116 °C and 121 °C autoclaved according to scheme B (*n* = 27), Figure S10: Concentrations of 2-KDG after heat sterilization of 10% glucose solutions at 111 °C, 116 °C, and 121 °C autoclaved according to scheme B (*n* = 27), Figure S11: Concentrations of 3-DG/3-DGal after heat sterilization of 10% glucose solutions at 111 °C, 116 °C, and 121 °C autoclaved according to scheme B (*n* = 27), Figure S12: Concentrations of 3,4-DGE after heat sterilization of 10% glucose solutions at 111 °C, 116 °C, and 121 °C autoclaved according to scheme B (*n* = 27), Figure S13: Concentrations of 5-HMF after heat sterilization of 10% glucose solutions at 111 °C, 116 °C, and 121 °C autoclaved according to scheme B (*n* = 27), Figure S14: Concentrations of GDPs in 10% (*w*/*v*) glucose solutions in PP bottles heat sterilized at 121 °C for 350 min (F0 = 202 min) (*n* = 9).
