# Supplementary material for: Quantification of Degradation Products Formed during Heat Sterilization of Glucose Solutions by LC-MS/MS: Impact of Autoclaving Temperature and Duration on Degradation
Source: Pharmaceuticals (Basel). 2021 Nov 1;14(11):1121. doi: 10.3390/ph14111121 (PMC8625795; doi:10.3390/ph14111121)

Figure S1. EZ-Tests® for the control of germ killing of the lead germ Geobacillus stearothermophilus after heat sterilization.

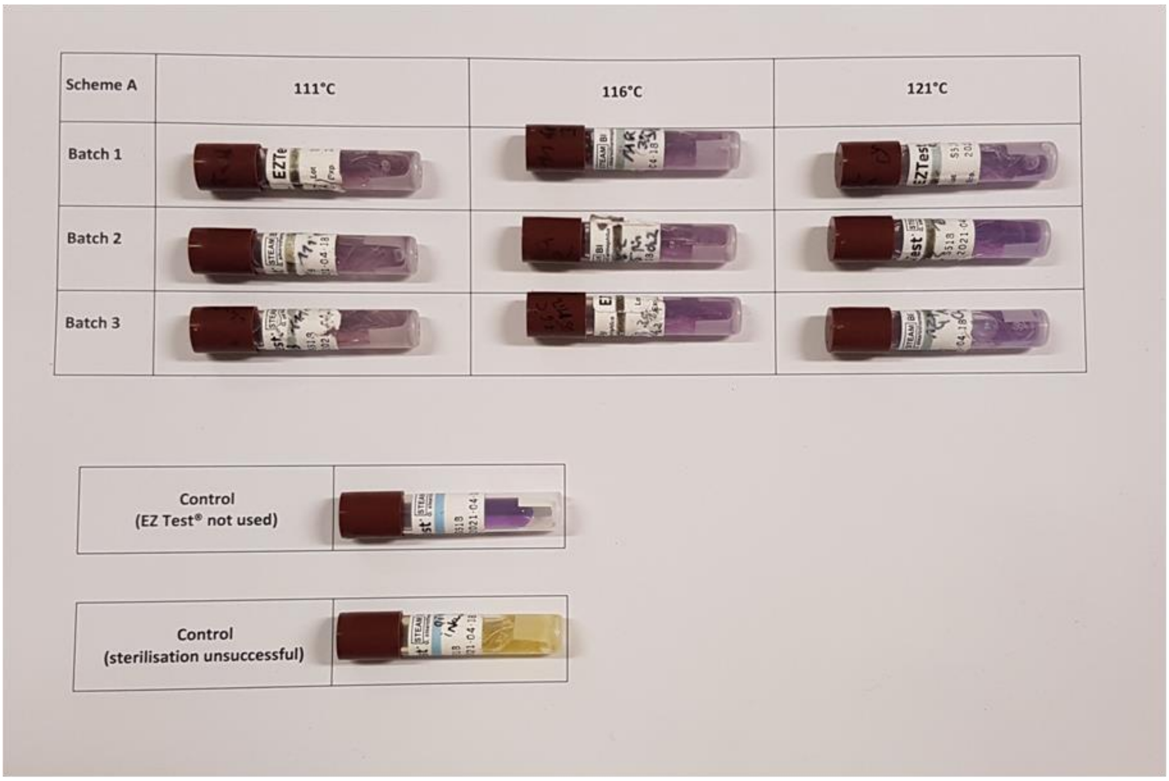

This figure shows the successfully performed heat sterilization at an F0 value suitable for sterility (according to Table 2 in the manuscript).

Graphical representation of the concentrations of degradation products in 10 % glucose solutions at different temperatures (111 °C; 116 °C and 121 °C) and different F0 times according to autoclaving schemes A and B.

Figure S2. Concentrations of GO after heat sterilization of 10 % glucose solutions at 111 °C, 116 °C and 121 °C autoclaved according to scheme A *(n=27)*.

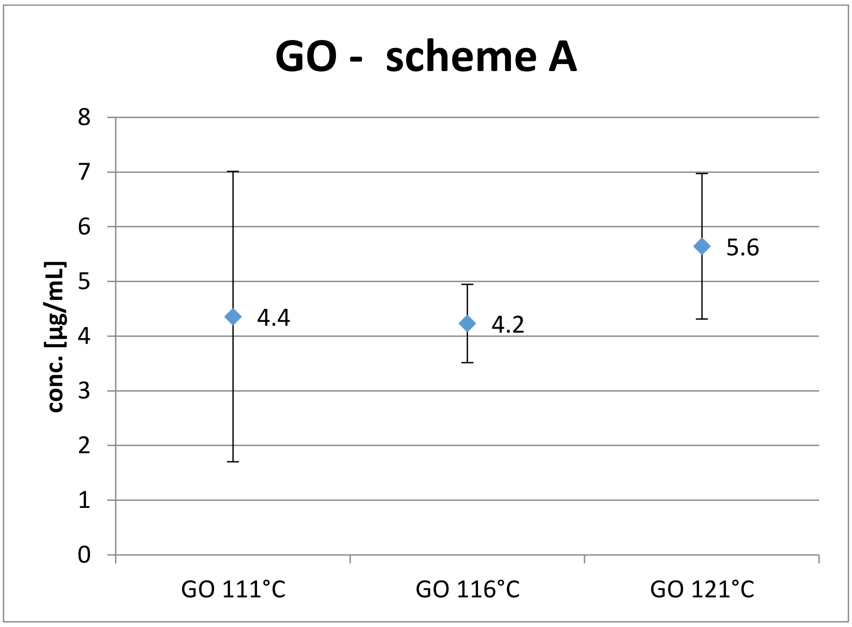

Figure S3. Concentrations of MGO after heat sterilization of 10 % glucose solutions at 111 °C, 116 °C and 121 °C autoclaved according to scheme A *(n=27)*.

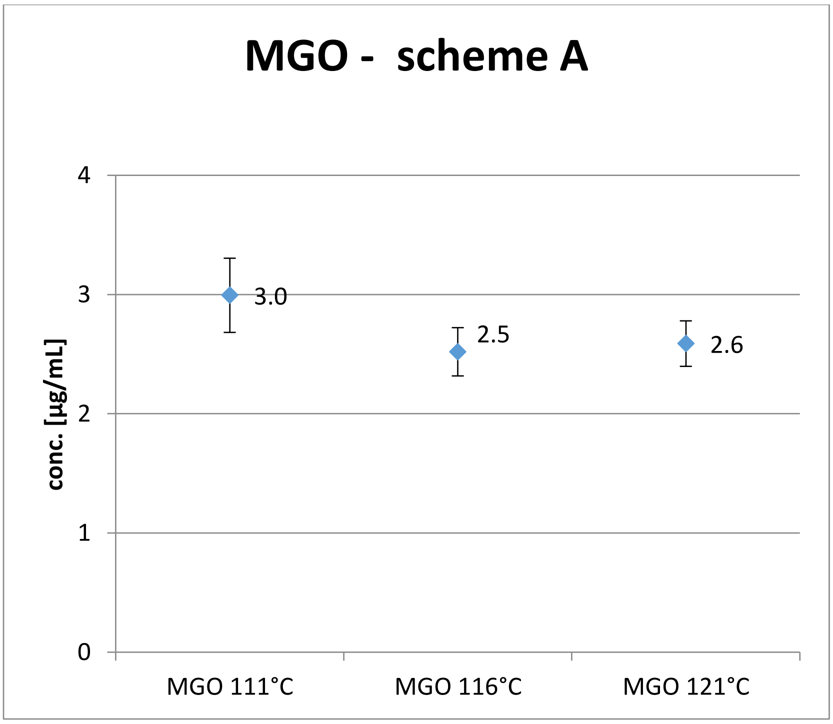

Figure S4. Concentrations of 2-KDG after heat sterilization of 10 % glucose solutions at 111 °C, 116 °C and 121 °C autoclaved according to scheme A *(n=27)*.

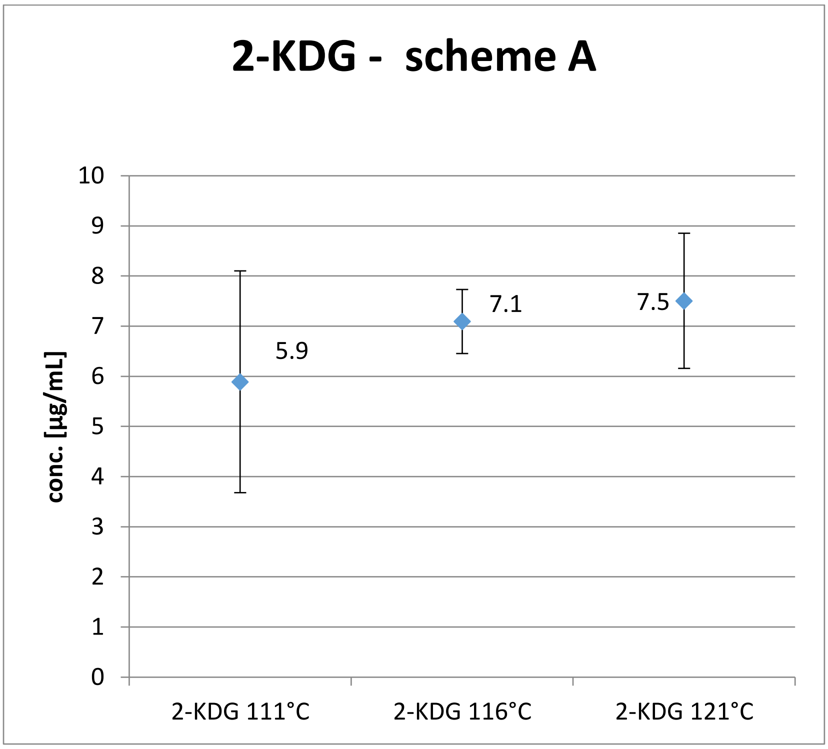

Figure S5. Concentrations of 3-DG/3-DGal after heat sterilization of 10 % glucose solutions at 111 °C, 116 °C and 121 °C autoclaved according to scheme A *(n=27)*.

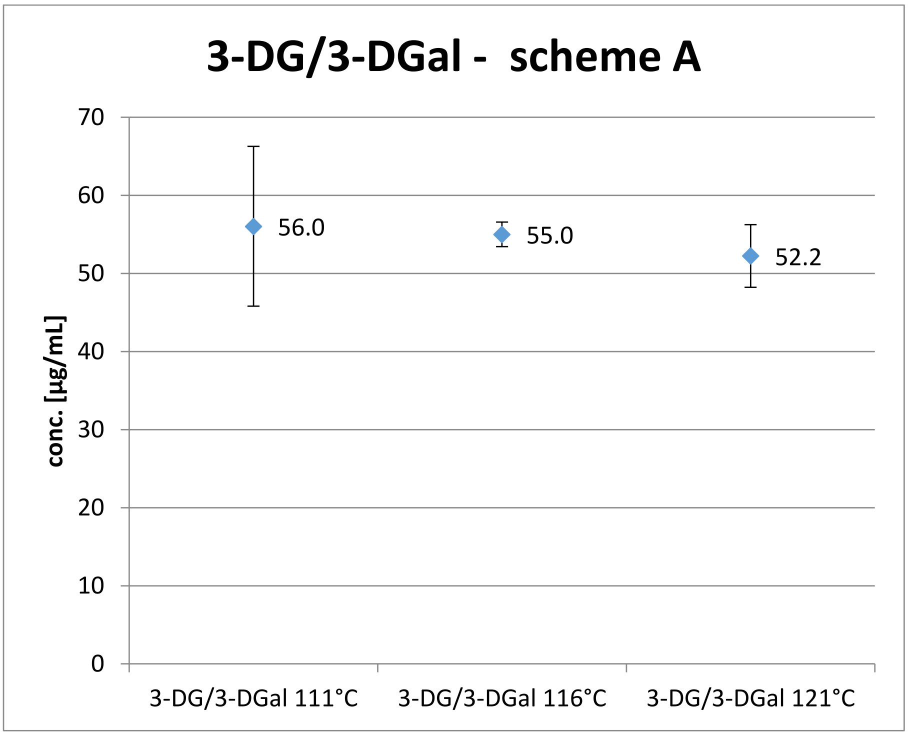

Figure S6. Concentrations of 3,4-DGE after heat sterilization of 10 % glucose solutions at 111 °C, 116 °C and 121 °C autoclaved according to scheme A *(n=27)*.

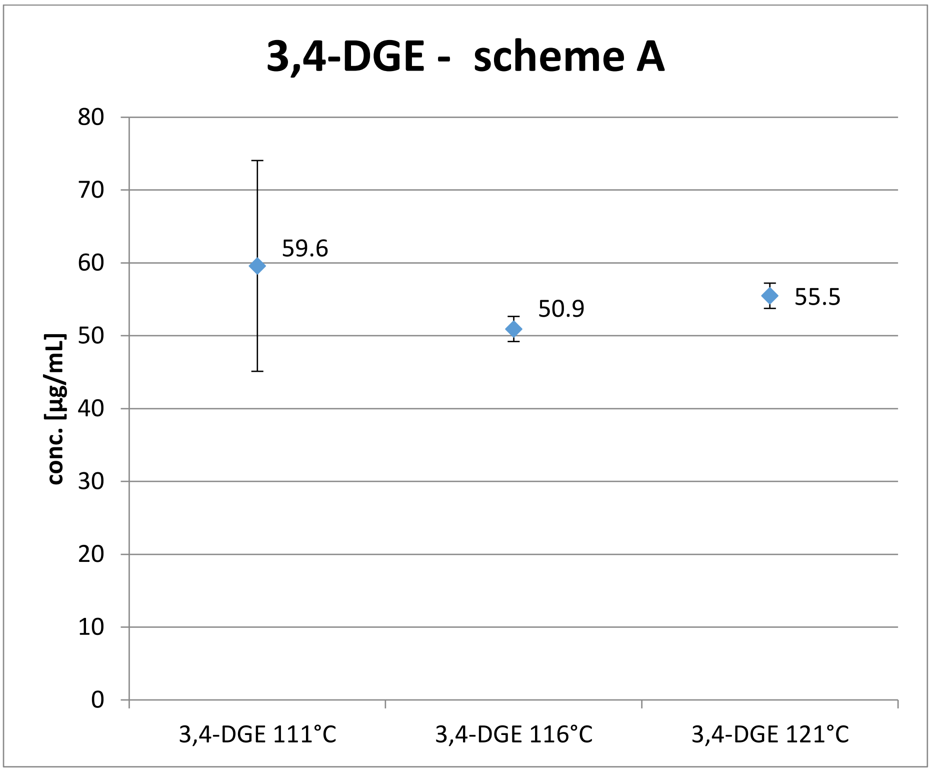

Figure S7. Concentrations of 5-HMF after heat sterilization of 10 % glucose solutions at 111 °C, 116 °C and 121 °C autoclaved according to scheme A *(n=27)*.

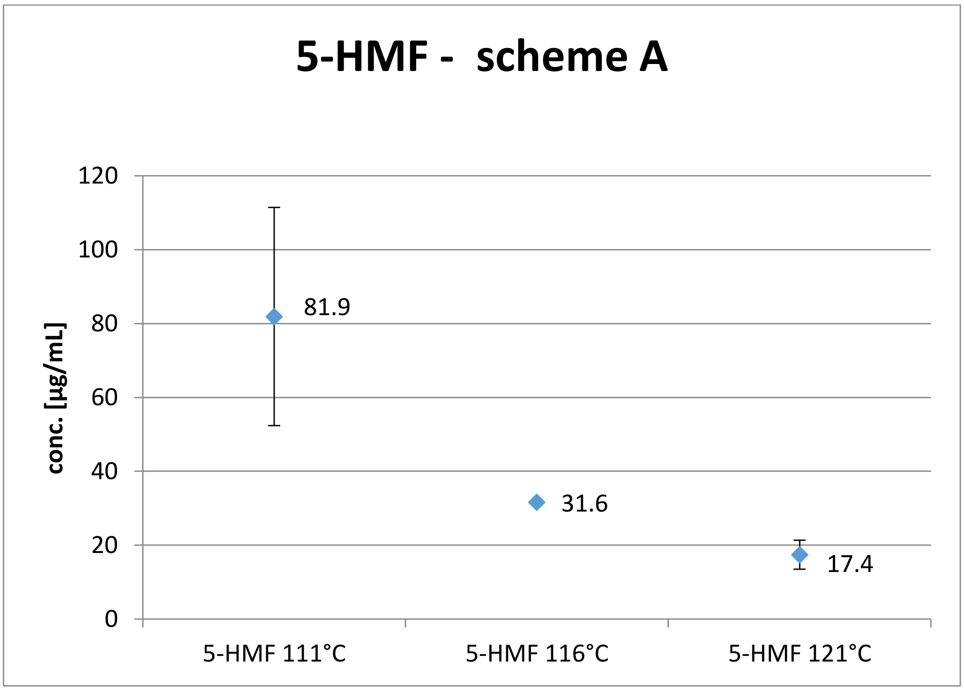

Figure S8. Concentrations of GO after heat sterilization of 10 % glucose solutions at 111 °C, 116 °C and 121 °C autoclaved according to scheme B *(n=27)*.

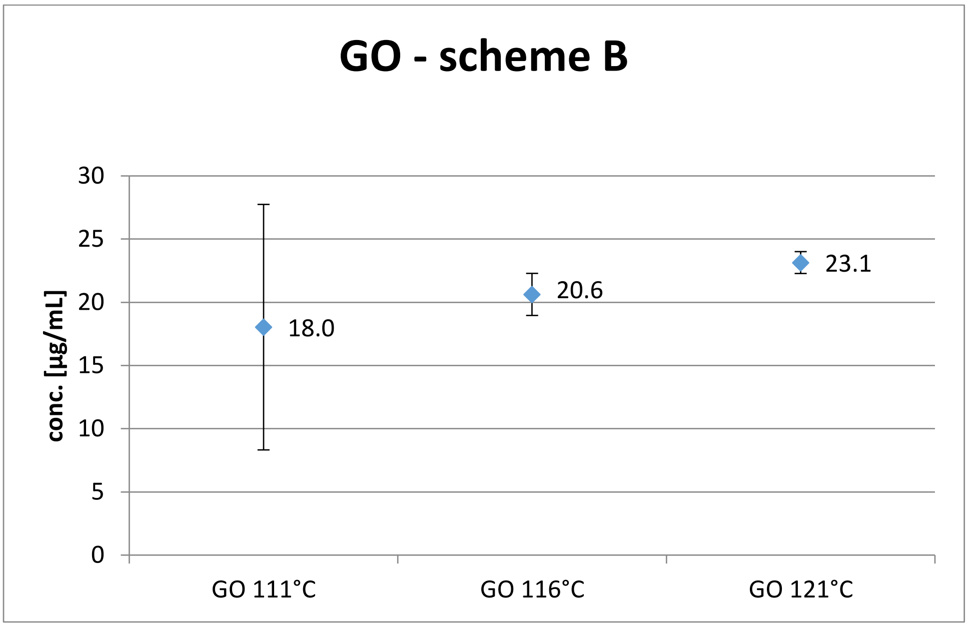

Figure S9. Concentrations of MGO after heat sterilization of 10 % glucose solutions at 111 °C, 116 °C and 121 °C autoclaved according to scheme B *(n=27)*.

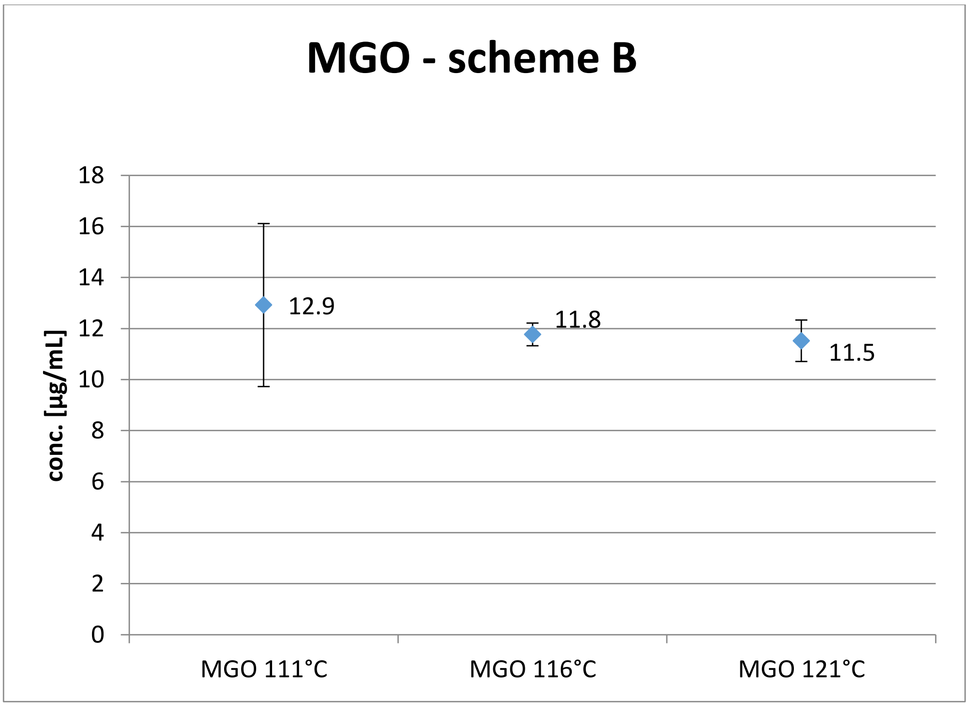

Figure S10. Concentrations of 2-KDG after heat sterilization of 10 % glucose solutions at 111 °C, 116 °C and 121 °C autoclaved according to scheme B *(n=27)*.

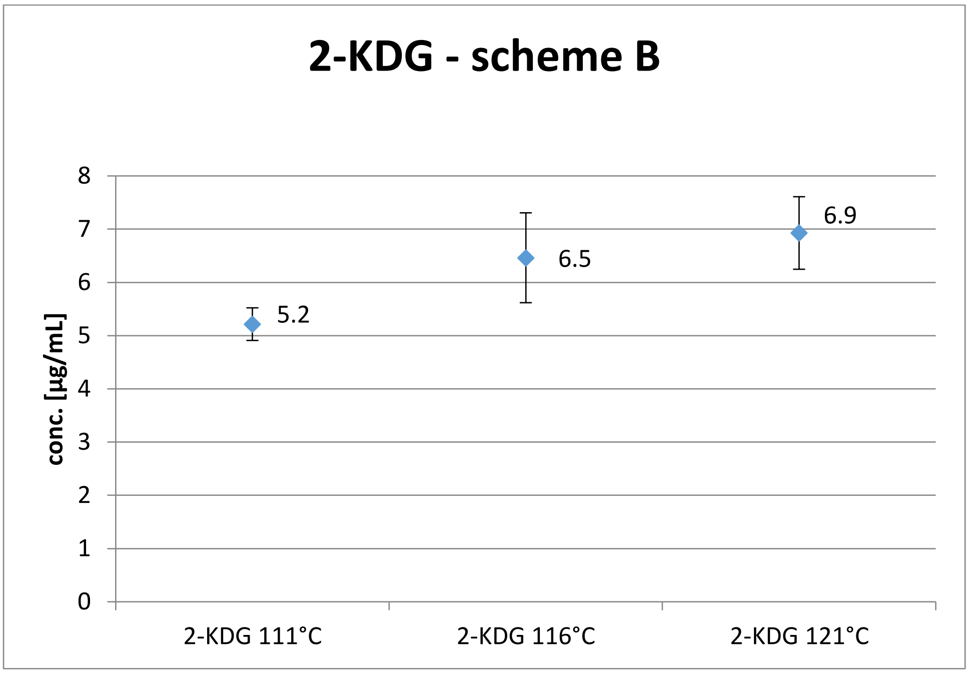

Figure S11. Concentrations of 3-DG/3-DGal after heat sterilization of 10 % glucose solutions at 111 °C, 116 °C and 121 °C autoclaved according to scheme B *(n=27)*.

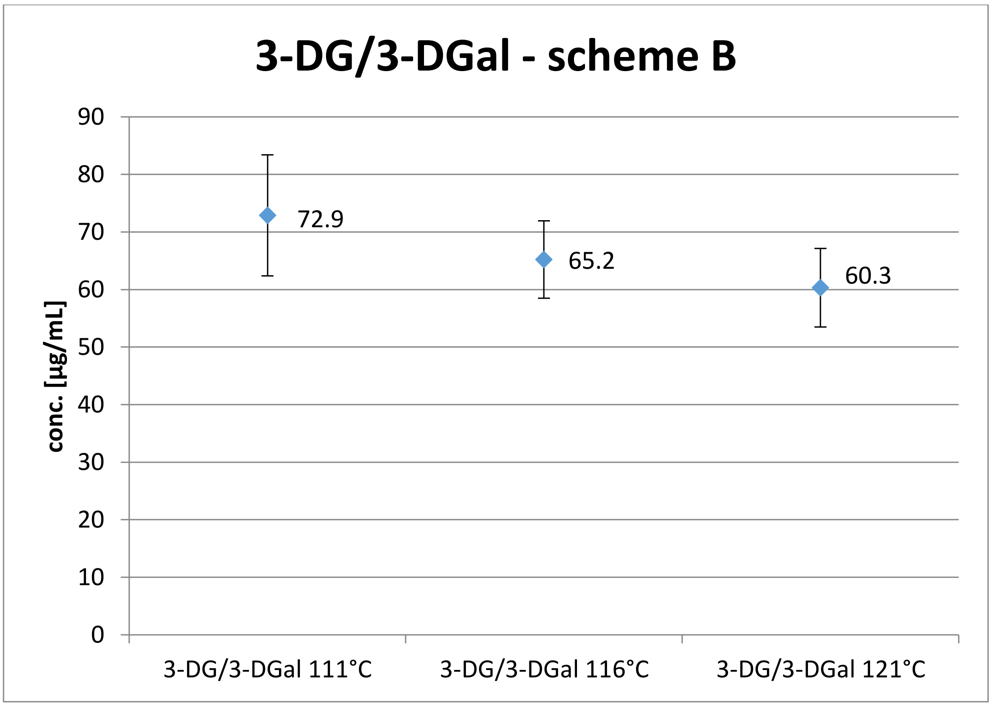

Figure S12. Concentrations of 3,4-DGE after heat sterilization of 10 % glucose solutions at 111 °C, 116 °C and 121 °C autoclaved according to scheme B *(n=27)*.

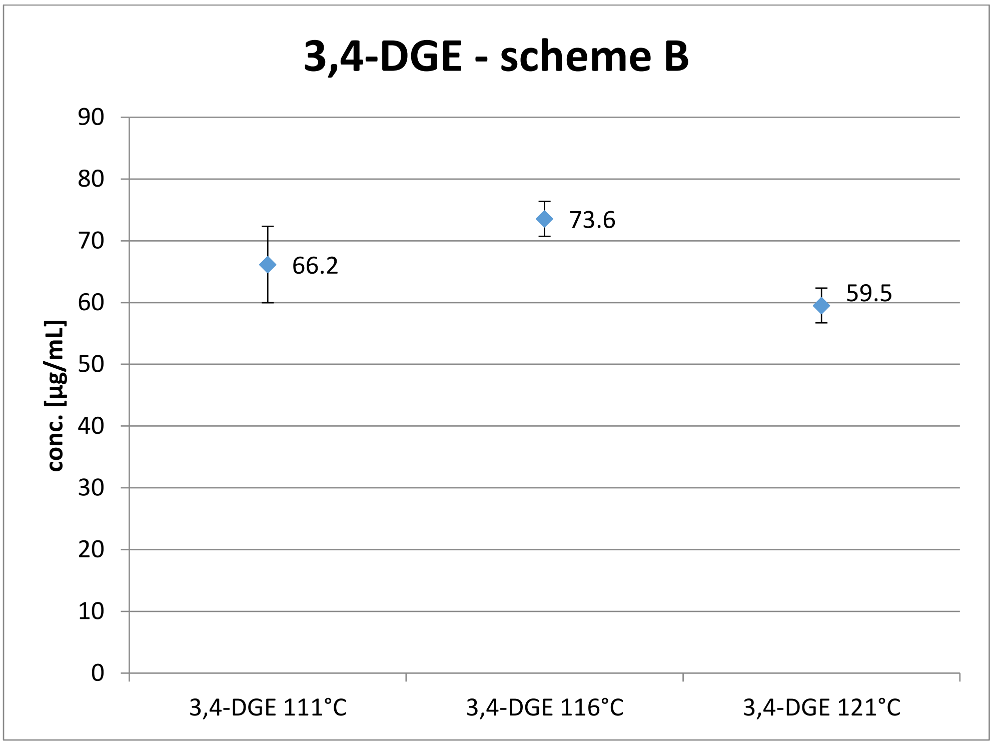

Figure S13. Concentrations of 5-HMF after heat sterilization of 10 % glucose solutions at 111 °C, 116 °C and 121 °C autoclaved according to scheme B *(n=27)*.

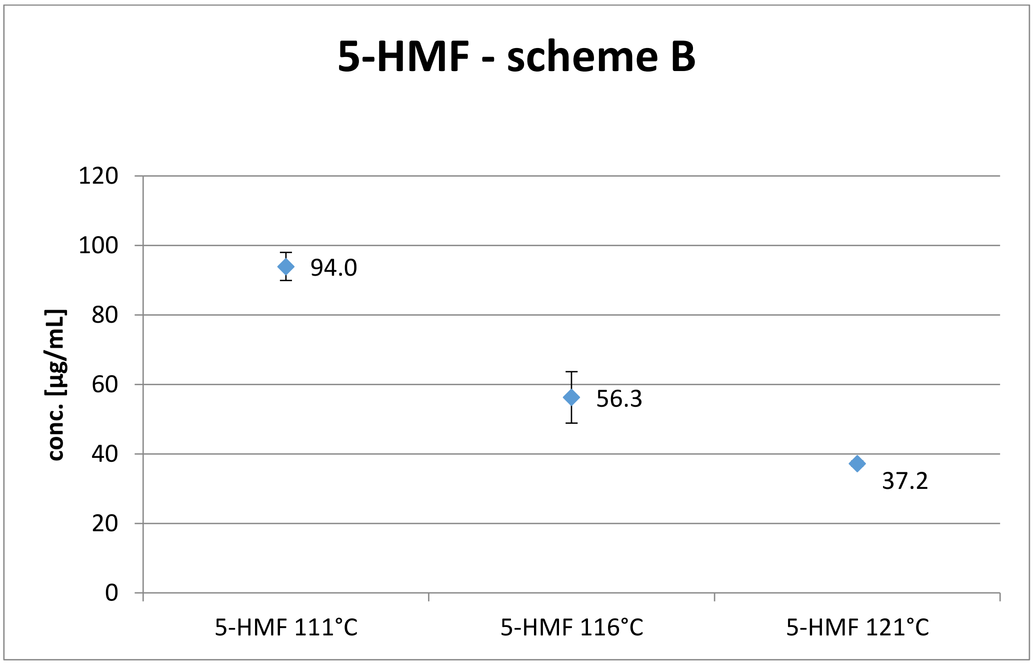

Figure S14. Concentrations of GDPs in 10 % (w/v) glucose solutions in PP bottles heat sterilized at 121 °C for 350 min (F0 = 202 min) *(n=9)*.

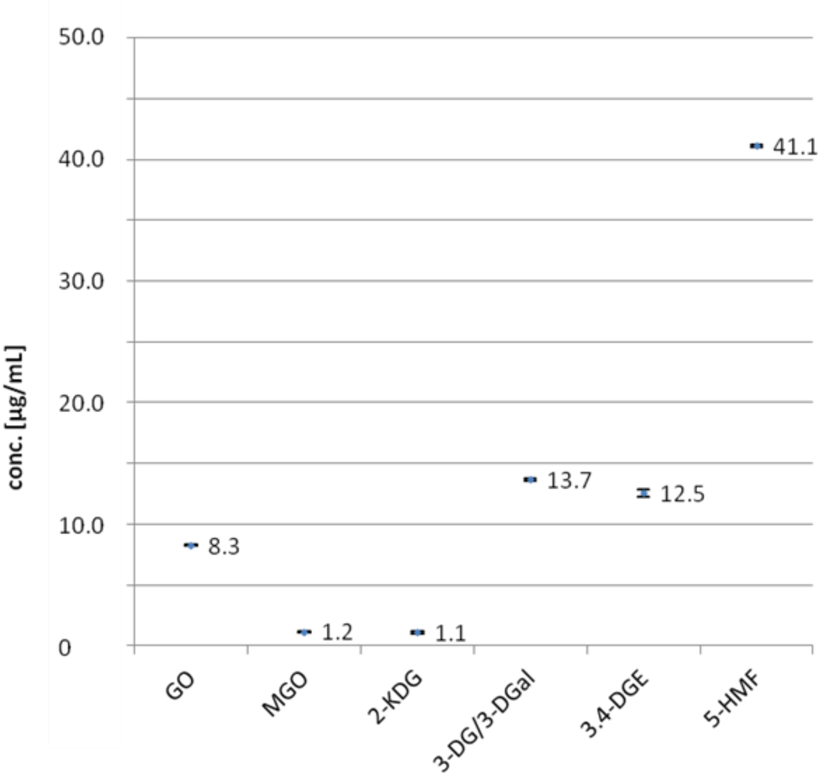

Supplement: Supplementary file 1 [file pharmaceuticals-14-01121-s001.zip › pharmaceuticals-1395378-supplementary.pdf]
